# Sprouts vs. Microgreens as Novel Functional Foods: Variation of Nutritional and Phytochemical Profiles and Their In vitro Bioactive Properties

**DOI:** 10.3390/molecules25204648

**Published:** 2020-10-12

**Authors:** Aneta Wojdyło, Paulina Nowicka, Karolina Tkacz, Igor Piotr Turkiewicz

**Affiliations:** Department of Fruit, Vegetable and Nutraceutical Plant Technology, Wrocław University of Environmental and Life Sciences, 37 Chełmońskiego Street, 51-630 Wrocław, Poland; paulina.nowicka@upwr.edu.pl (P.N.); karolina.tkacz@upwr.edu.pl (K.T.); igor.turkiewicz@upwr.edu.pl (I.P.T.)

**Keywords:** polyphenols, amino acids, carotenoids, chlorophylls, L-ascorbic acid, organic acids, sugars, anti-oxidant, anti-diabetic, anti-obesity

## Abstract

The aim of the study was to analyze potential health-promoting and nutritional components (polyphenols, L-ascorbic acid, carotenoids, chlorophylls, amino acids, organic acid, sugars, ash and pectins) of selected sprouts (radish, lentil, black medick, broccoli, sunflower, leek, beetroot, mung beans) and microgreens (kale, radish, beetroot, green peas, amaranth). Moreover, antioxidant capacity (2,2′-azino-bis(3-ethylbenzothiazoline-6-sulphonic acid) (ABTS), ferric reducing ability of plasma (FRAP), and oxygen radical absorbance capacity (ORAC)), in vitro anti-diabetic potential (inhibition of α-amylase and α-glucosidase), and anti-obesity (pancreatic lipase) and anti-cholinergic (acetylcholinesterase and butyrylcholinesterase) activity were evaluated. The results of this study show that sprouts are effective in antioxidant capacity as a result of a high content of polyphenols and L-ascorbic acid. Additionally, sprouts are better sources of amino acids, pectins and sugars than microgreens. Microgreens were characterized by high content of carotenoids and chlorophylls, and organic acid, without any sugars, exhibiting higher anti-diabetic and anti-cholinergic activity than sprouts. Some selected sprouts (broccoli, radish, lentil) and microgreens (radish, amaranths, kale) should be used daily as superfoods or functional food.

## 1. Introduction

Germination is a complex stage of plant ontogenesis involving growth initiation but not comprising final growth processes and maturation [1,2]. The gist of germination is restoring metabolism of dormant seeds not showing any physiological activity. The process activates the seed embryo and allows for seedling growth [3,4,5]. Seven- or ten-day-old sprouts are of appropriate size for harvest, allowing for post-harvest handling and commercialization. The material shows higher content of phytochemicals than other vegetables [2].

Microgreens are immature plants produced from the seeds of vegetables, cereals or herbs. They are 5 to 10 cm long and comprise a stem and cotyledons. They are usually harvested at the base of their cotyledons, just after the cotyledons emerge but before true leaves develop. This takes place from 7 to 21 days after germination, depending on the species [6,7,8,9,10]. The life cycle of microgreens is very short, and they quickly deteriorate after harvest. When stored at room temperature, they can be safely consumed within 1 to 2 days [9]. Contrary to sprouts, the term “microgreens” is not scientific; rather, it is used for marketing purposes. Their production began at the end of 1980s [6], and in recent years, they have been constantly gaining popularity due to growing interest in functional foods [10].

Germinating seeds could contain from 2 to 10 times more phytochemicals as compared with commercial adult plants. This content depends on the species; cultivar; environmental conditions; and the time of germination, storage, and processing [10]. So far, sprouts have been more often than microgreens recognized as wellness and health-promoting foods, widely recommended by dietitians due to their high content of nutrients and bioactive compounds, such as flavonoids, hydroxycinnamic acids, vitamins and glucosinolates, minerals, and carotenoids [4,10]. These phytochemicals seem to play a crucial role in protecting the human body against different types of chronic disorders such as cardiovascular diseases, diabetes, and cancer [10,11]. Additionally, sprout and microgreen leaves are characterized by a low calorific value (29–128 kcal/100 g) and low glycemic index [10,11].

The number of species that can be consumed as sprouts or microgreens is huge. Their seeds differ in germination rate, taste and chemical composition. The most popular seeds used for the production of sprouts and microgreens include those of cereals, legumes, oilseeds or crucifers, e.g., lentils, soybean, broccoli, alfalfa, radish, sunflower, cress, pumpkin, mung bean or onion (chives) [1].

Nowadays, in a society that is more aware of and interested in healthy lifestyles and prevention of diseases, sprouts and microgreens seem highly desirable products. Apart from offering the mentioned health benefits, they can be easily and quickly produced and used in many different ways. Nevertheless, sprouts and microgreens are still considered innovative culinary ingredients. They are used as additions to sandwiches, salads, soups, desserts and drinks [4,10,12], and their popularity is due to their delicate texture, unique colors and high palatability, making them useful in the culinary industry. The Japanese market offers many kinds of foodstuffs based on sprouts or microgreens in the form of powder or additives to alcohols, juices, and teas [13]. They are also available all year round, even in the winter with its lack of fresh fruit and vegetables [10].

Nowadays, with aging populations increasingly often suffering from major chronic diseases of the 21st century, such as obesity, cardiovascular diseases, cancers, and type 2 diabetes, a diet rich in fruit and vegetables is often recommended as a preventive measure. All organizations with focus on nutrition (i.e., WHO, USDA from USA or IŻŻ from Poland) claim that diets rich in fruits and vegetables provide abundant compounds of known protective benefits against chronic diseases. These compounds include polyphenols, vitamins (i.e., L-ascorbic acid or carotenoids as provitamin A precursors), amino acids, or chlorophylls. It is therefore crucial to identify the health benefits of commercial sprouts and microgreens that are commonly consumed in the spring when fresh fruits, vegetables and herbs are less readily available. Studies on the relationship between chemical composition and biological activity of sprouts and microgreens are limited, yet these products are commonly offered in day to day sale.

Therefore, the aim of this work was to characterize and compare natural antioxidants (L-ascorbic acid, phenolic compounds and carotenes) and their in vitro biological activity (antioxidant capacity, anti-diabetic, anti-obesity and anti-cholinesterase activity) in sprouts and microgreens to foster their application as natural, healthy foods. An additional aim was to characterize nutritional values of sprouts and microgreens in terms of their content of amino acids, pectins, ash, sugar, organic acids and soluble solids. We also determined their dry matter and pH. Some of the selected sprouts and microgreens were investigated for the first time.

## 2. Results and Discussion

### 2.1. Basic Chemical Composition of Sprouts and Microgreens

The basic chemical composition of the tested sprouts and microgreens is presented in Table 1. The sprouts and microgreens differed in their chemical composition (*p* < 0.05).

Mean dry matter content ranged from 4.7 (mung bean) to 46.4% (lentil) for sprouts, and from 4.1 (kale) to 8.1% (green pea) in microgreens. This was overall similar to the values previously reported by Xiao et al. [12]. Lower dry matter content probably reflected more advanced vegetative growth attained by the microgreens. High content of water in microgreens and some sprouts (black medick, sunflower, beetroot and mung bean) indicated high water storage capacity and adaptations preventing water loss.

The tested sprouts and microgreens showed low content of soluble solids (SSC). This was especially true for microgreens represented by kale, radish, beetroot, and amaranths, and for sprouts represented by black medick, sunflower or leek. To date, there have been no reports on SSC in sprouts and microgreens, however, their content seems to be lower than in artichoke [14] or other vegetables. The content of sugar was only examined in sprouts of lentil, broccoli and radish (up to 1.8 g/100 g fresh weight fw). The main identified saccharide was fructose, with glucose and saccharose taking the second and third place, respectively. Sorbitol, mannose and rhamnose occurred only in leek, broccoli and lentil, respectively. The lowest sugar content was detected in black medick and mung bean (>0.2 g/100 g fw) sprouts, while in beetroot sprouts, similarly to microgreens, no sugars were identified. Our results corroborated those obtained for sea buckthorn fruits [15] and artichoke [14], but differed from those for microgreens analyzed by Choe et al. [10]. Apart from their growth-related functions, sugars also serve as signaling molecules that modulate light signaling pathways. Moreover, intense sucrose accumulation in the leaves is associated with a reduction in nitrates or decreased photosynthetic rate [16].

Sprouts and microgreens were characterized by high pH (>5.3), and titratable acidity lower than 0.7 and 0.4 g/100 fw, respectively. The content of organic acid was significantly higher for microgreens than for sprouts. In our study, oxalic acid predominated over the other organic acids, but its levels were 10 times higher in microgreens than in sprouts. The remaining organic acids, i.e., citric, malic or quinic and succinic acids, were at similar levels in both materials (Table 1). These trends were in agreement with those reported by Silva et al. [3]. Low amounts of phytic acid were detected only in black medick, sunflower and beetroot. Apart from different aromatic compounds, sugars, organic acids and pH are crucial for organoleptic characteristics as well as acceptability, quality and nutritional values and microbial stability. Organic acids are known for their both beneficial and adverse effects on human health. For example, citric and malic acids support the action of antioxidants due to their ability to chelate metal ions, i.e., copper and iron that catalyze adverse oxidation processes. Phytic acid affects the uptake of micronutrients in the digestive tract [17]. On the other hand, high content of oxalic acid salts is unfavorable, because they can form hardly soluble crystals of calcium oxalate in various organs, including atherosclerotic plaques in the coronary arteries. Oxalic acid is known to reduce mineral bioavailability from food. High content of oxalic acid in microgreens is the effect of postharvest treatment with this chemical as an effective method for preserving the food against bacteria and pathogens.

Additionally, plant treatment with oxalic acid significantly delays quality deterioration during storage. The acid specifically reduces yellowing of the leaves by stopping chlorophyll degradation, slowing down the respiration rate, electrolyte leakage, and ammonia production [18]. In recent years, many authors have reported on the beneficial effects of applying oxalic acid on artichokes [19] or rocket and baby spinach leaves [18].

Pectins are among the most complex components of plant cell walls. Depending on their molecular composition and chemical configuration, pectin matrices can change mechanical behavior of plants [20] in terms of their elasticity, hardness or fibrousness. Unfortunately, the analyzed sprouts and microgreens featured low content of pectins, except for lentil sprouts. From the human perspective, pectins play an important health-promoting role by impacting metabolic and physiological processes of the organism [20].

Mineral elements are important for human health, and their adequate daily intake is necessary for a good condition and wellness of the body [9]. Some authors [9,10] suggested that microgreens are a perfect source of minerals. No significant differences between sprouts and microgreens were detected, but the content of ash was higher in microgreens, especially for amaranths and kale (Table 1). According to previous studies, sprouts, microgreens or “baby” vegetables at early growth stages are better sources of nutrients than their mature counterparts [21,22]. The content of ash depends on the agrotechnical techniques and growing conditions, such as soil and climatic factors, exposure to light, and the type of species or varieties [22].

### 2.2. L-ascorbic Acid Content in Sprouts and Microgreens

Significant differences were found between the content of L-ascorbic acid in microgreens and sprouts, as shown in Table 1. Sprouts of broccoli (114.0 mg/100 g fw) and radish (94.0 mg/100 g fw) accumulated the greatest amounts of L-ascorbic acid, while its level in all microgreens fell below 32 mg/100 g fw. According to literature data [23], L-ascorbic acid is absent in unsprouted seeds of soybean, buckwheat, and mung bean, but its level increases significantly during germination and sprouting. The content of L-ascorbic acid in sprouts and microgreens was similar to that reported by Marton et al. [17] and Xiao et al. [12], respectively. Its levels in coriander (13.09 mg/100 g), cress and purple basil (73.27 and 98.04 mg/100 g fw) [4] were similar to those in the investigated microgreens.

Literature reports show that the content of L-ascorbic acid in sprouts and microgreens is not very high [12]. It may depend on multiple factors, such as cultivar, cultivation method, biotic and abiotic stress, harvest date and storage, access to light, water and nitrogen fertilizers [4]. Another essential factor is the time of germination, as the most intense synthesis of L-ascorbic acid occurs within a few days after sowing, and then its content is maintained at a constant level [12,17]. In some sprouted seeds (soybean and buckwheat), the initial peak was followed by a decrease in L-ascorbic acid content until reaching its original values [23]. This may explain why microgreens exhibited low levels of this compound. It should be remembered that regular consumption of vitamin C helps in diminishing oxidative stress, limiting undesirable reactions of enzymatic oxidation, and is necessary for collagen synthesis in humans [4,12,23].

### 2.3. Chlorophyll and Carotenoid Contents in Sprouts and Microgreens

The color of sprouts and microgreens is one of the first attributes that affects consumers’ choice and acceptability of the product, and, thus, it is considered an important parameter determining their quality. The results regarding identification and quantification of chlorophylls and carotenoids (carotene and xanthophylls) analyzed by UPLC-PDA-Qtof-ESI-MS system are summarized in Table 2. Fourteen major compounds, of which eight belong to chlorophylls, were identified in sprouts and microgreens. In general, microgreens were richer in chlorophylls and carotenoids than sprouts.

The content of chlorophylls in microgreens ranged from 195.6 to 638.5 μg/g fw and decreased in the following order: amaranth > green peas >> beetroot > radish > kale. The content of chlorophylls in sprouts oscillated from 6.0 to 108.5, with the highest value recorded for lentil. Previous studies [4] reported similar levels of chlorophylls (118.0–715.1 μg/g fw) in variable microgreens, such as mustard, pack choi, radish, or green and purple basil. The major compounds in the analyzed samples were chlorophylls (*a, a’*, and *b*), but some compounds, e.g., pheophytin (*a’*, *b* and *b’*), were not evaluated in sprouts. Both chlorophyll *a* and *b* and chlorophyll isomers *a′* and *b′,* and pheophytin *a′* are the major chlorophylls present in green plants [24]. The primary photosynthetic pigment is chlorophyll *a*. Chlorophyll *b* is not necessary for photosynthesis to occur, and, therefore, not all cells that perform photosynthesis contain chlorophyll *b*. An increase in the content of chlorophyll *b* results from adaption to shade, as it allows a plant to absorb a broader range of light wavelengths. Light is not only crucial for plant growth and development, but it also plays an important role in enhancing cellular metabolism and synthesis of defense-related secondary metabolites [25]. Sprouts and microgreens are very often grown in special, controlled environment growth chambers with artificial light sources [26]. This may explain why in all the analyzed samples, chlorophyll *a* predominated over chlorophyll *b*. The chlorophyll *a*:*b* ratio reflects the conditions in which sprouts and microgreens were cultivated. A high ratio indicates that a sample was exposed to the sun or intense light, as the high level of chlorophyll *b* is responsible for the more yellow color. In microgreens, this ratio was at a similar level (1.8–1.9), but in sprouts, chlorophyll *a* was up to 8.8 times more abundant than chlorophyll *b*, which meant than sprouts were richer in chlorophyll *a* than *b*, and the green color was more intense. Kyriacou et al. [4] determined the chlorophyll *a:b* ratio in mibuna and radish microgreens, which reached 2.85 and 4.20, respectively.

Yellow-orange fruits and green leafy vegetables are generally considered the richest source of carotenoids. Our study demonstrated an increase in carotenoid levels at germination and sprouting time, which makes sprouts and microgreens moderately good sources of these compounds. The content of carotenoids followed the same pattern as the content of chlorophyll (Table 2). In microgreens, it ranged from 1510.1 to 4073.5 μg/g fw and decreased in the following order: amaranth > kale > green pea > beetroot > radish. The content of carotenoids in sprouts was more variable and ranged from 22.5 (mung bean) to 948.8 (radish) μg/g fw. Major compounds in the analyzed sample included oxygenated carotenoids, such as xanthophylls (lutein, neo- and zeo-xanthin) and carotenes *α+β*. The analyzed microgreens were richer in (*α+β*)-carotene, lutein, zeo- and neo-xanthin and neochrome than sprouts. According to the literature, plant parts exposed to sun or light generally accumulate significantly greater amounts of xanthophyll and *β*-carotene and trace amounts of *α*-carotene. *α*- and *β*-carotene and cryptoxanthins are precursors of provitamin A [27], and, therefore, their levels were calculated. The content of provitamin A in sprouts was approximately 21 times lower (*p* < 0.05) than in microgreens. The content of provitamin A in microgreens ranged from 281.6 to 698.6 μg RAE (recommended dietary allowance) [27], which means that a diet containing radish or kale helps in meeting daily dietary recommendations for this compound. Daily carotenoid intake in adults in European countries, e.g., Spain, reaches 9.54, but in France, it is up to 16.06 mg [27].

Carotenoids play a major role in the protection of plants against photooxidative processes [25] and in the protection of humans against cancers of, e.g., liver, colon, lung, pancreas or prostate [10,28]. They are also efficient antioxidants [22,28]. Consumption of carotenoid-rich products has been demonstrated to bring health benefits in the form of ameliorating degenerative and cardiovascular diseases [7,10,29].

### 2.4. Polyphenol Content in Sprouts and Microgreens

UPLC-PDA-FL analysis of the methanolic extracts of sprouts and microgreens revealed their phenolic composition (Table 2). Polyphenol profiles differed significantly across sprout and microgreen samples, and variations were observed with respect to major phenolic components. All samples contained some flavan-3-ols (catechins and polymeric procyanidins), phenolic acids (mainly caffeoylquinic, ferulic, sinapic, chlorogenic and their derivatives), flavonols (mainly quercetin, kaempferol, isorhamnetin), and flavones (daidzein and genistein). Some samples (radish, beetroot, amaranth) comprised also anthocyanins, mainly cyanidin derivatives. Phenolic acids, such as caffeoylquinic, ferulic, sinapic, or chlorogenic acid and their derivatives, were common in all investigated samples. Dominant plant flavonols include derivatives of quercetin, kaempferol, and isorhamnetin [2,8]. Sprouts were richer in total polyphenols (26.7–191.1 mg/100 g fw; especially radish, lentil, broccoli, and sunflower) than microgreens (23.3–132.9 mg/100 g fw; especially amaranth and green pea).

Phenolic acids were a dominant group of polyphenols only in sprouts of radish, broccoli and sunflower (55, 60, 68% of total phenolic compounds, respectively) and in microgreens of green pea and amaranth (23 and 32% of total phenolic compounds, respectively). Lentil sprouts were rich in polymeric procyanidins and isoflavones, and broccoli sprouts additionally in monomeric flavan-3-ols and polymeric procyanidins. Anthocyanins were quantified in radish and beetroot sprouts and microgreens, and in amaranth microgreens, they accounted for 35% of total phenolic compounds. As mentioned above [2,4], microgreens contained only scant amounts of flavonols and their glycosides that are common in mature green vegetables, which suggests significant phenolic transformation during plant ontogenesis. L-phenylalanine is converted into *trans*-cinnamic acid by phenylalanine ammonia lyase during synthesis of phenolic compounds. It can be therefore expected that sprouts and microgreens are rich in phenolic acid and flavan-3-ols that are primary compounds synthesized via the phenylpropanoid metabolic pathway. The sprouts of cress are particularly rich in flavonoids, and of radish in phenolic acids, while lentil sprouts are less abundant in these compounds [17].

Samotyja et al. [1] determined polyphenol content in the sprouts of five species (mung bean, radish, sunflower, wheat and lentil) and found their highest levels in sunflower and radish. The content of polyphenolic compounds doubles in germinated seeds of quinoa, buckwheat and wheat as compared with their seed levels. Most of these compounds were found in buckwheat seeds and sprouts. Kyriacou et al. [4] examined 13 microgreens belonging to *Lamiaceae*, *Brassicaceae*, *Malvaceae*, *Chenopodiaceae*, and *Apiaceae* species, and found green and purple basil microgreens (*Lamiaceae*) to be the richest in phenolic compounds. Additionally, they identified alternative phenolic-rich microgreens from the *Apiaceae* family. Cevallos-Casals and Cisneros-Zevallos [5] compared polyphenolic content in the seeds and sprouts of 13 different crops (broad bean, sunflower, soybean, mung bean, radish, fenugreek, broccoli, lentil, wheat, kale, mustard, alfalfa and onion), and found the sprouts of mustard, sunflower and broccoli the most abundant in these compounds. A study by Silva et al. [3] demonstrated that the sprouts of alfalfa, soybean and mung bean are a rich source of numerous bioactive compounds with antioxidant properties (phenolic acids, flavones, flavonols, isoflavones, monoterpenes) that may inhibit or delay cell degeneration and damage caused by oxidative stress.

### 2.5. Amino Acid Content in Sprouts and Microgreens

This work presents a qualitative and quantitative analysis of free amino acids in the sprouts and microgreens performed using a derivatization method with AQC and LC-PDA-QTof-ESI-MS operated in negative ionization mode. We analyzed 24 amino acids of variable abundance as present in Table 3. Two the most abundant free amino acids in sprouts and microgreens included L-asparagine (particularly high content in lentil, black medick, and leek sprouts and radish and green bean microgreens), and L-glutamine (particularly high content in radish, broccoli, sunflower, leek, and beetroot sprouts, and in kale and amaranth microgreens). The content of L-ornithine (only characteristic of lentil sprouts and kale microgreens), L-proline, L-methionine (not detected in beetroot sprouts and microgreens and amaranth microgreens), L-homocysteine (only characteristic of black medick sprouts and kale microgreens) in sprouts was always very low (<1%). Additionally, L-cysteine was detected in microgreens, but L-proline was absent in these samples.

The sum of free amino acids was generally higher in sprouts and ranged from approximately 162.3 (radish) to 455.0 (beetroot) mg/100 g fw for microgreens, and from 321.4 (beetroot) to 1292.1 (lentil) mg/100g fw for sprouts (Table 3).

The concentration of amino acids in radish or beetroot sprouts and microgreens varied significantly, and might, as suggested by Silva et al. [30], result from metabolic changes during growth and ripening. According to Tarasevičiene et al. [31], amino acid levels in broccoli were growing for up to 72 h of germination and then stabilized to remain very similar after 120 h. These authors [31] indicated that changes in amino acid content during germination could be related to protein hydrolysis, synthesis, and re-arrangement that involved mobilization of protein reserves in cotyledons and synthesis of new proteins necessary for the sprout growth. Furthermore, the amino acids produced by hydrolysis of the protein reserves are not used solely for the synthesis of new components but may also be used as energy sources, especially at early stages of germination.

Generally, even though the content of amino acids in sprouts and microgreens is low, they are still important sources of these compounds, as some amino acids cannot be produced by the human body and need to be supplied with food (e.g., L-histidine, L-isoleucine, L-leucine, L-lysine, L-methionine, L-phenylalanine, L-threonine, L-tryptophan, and L-valine). The content of these amino acids ranged from 56.3 (beetroot) to 248.2 (broccoli) mg/100g fw in sprouts and from 24.6 (beetroot) to 83.7 (radish) mg/100 g fw in microgreens (Table 3). Apart from nutritional properties, amino acids also affect taste and flavor, as a number of them have a distinctively bitter taste (e.g., tyrosine, arginine, leucine, valine, methionine, and histidine). Additionally, methionine and L-homocysteine, as amino acid thiols, play an important role in abiotic stress tolerance in plants, and they are the most important antioxidants that protect cells from oxidative damage [32].

### 2.6. Biological Properties of Sprouts and Microgreens and Their Anti-Oxidant, Anti-Diabetic, Anti-Obesity and Anti-Cholinergic Activity

Sprouts and microgreens are rich in natural antioxidants, including phenolic compounds, carotenoids, chlorophylls, ascorbic acid, etc., that determine their considerable antioxidant capacity. Antioxidant properties were assessed using (2,2′-azino-bis(3-ethylbenzothiazoline-6-sulphonic acid) (ABTS), ferric reducing ability of plasma (FRAP), and oxygen radical absorbance capacity (ORAC)) assays. As shown in Table 4, sprouts accumulate significantly higher amounts of oxygen radical scavengers than microgreens. ORAC capacity in sprouts was the highest in beetroot and descended in the following order: beetroot, broccoli > radish, lentil >> black medick, sunflower > leek, mung bean, while in microgreens, this order was as follows: beetroot >> kale > radish > amaranth > green pea.

A similar tendency was observed for scavenging radical ABTS and reduced ferrous ion (Fe^+3^) in the FRAP assay. Overall, the antioxidant capacity was higher in sprouts than in microgreens, with a notable exception of mung bean sprouts. The antioxidant capacity measured for mung bean sprouts was below 0.01 mmol Trolox/100 g fw. Mung bean sprouts had low content of polyphenols, chlorophylls and L-ascorbic acid. Previous works [5,15,33] reported that antioxidant potential can be related to the content of phenolics and other bioactive compounds. Antioxidant capacity measured by ABTS and FRAP assays correlated with the content of L-ascorbic acid (r^2^ = 0.671 and 0.872); polyphenols (r^2^ = 0.682 and 0.538), particularly flavan-3-ols (r^2^ = 0.742 and 0.649); and polymeric procyanidins for the ABTS assay (r^2^ = 0.758). For the ORAC test, the strongest correlations were determined for polyphenols (r^2^ = 0.327), flavan-3-ols (r^2^ = 0.481) and L-ascorbic acid (r^2^ = 0.438). Other bioactive compounds, such as volatiles and organic acids found in the sample, should not be ignored, as they may contribute to the obtained results. Silva et al. [3] mentioned that volatile compounds or organic acids present in the sprouts were reported as antioxidants, and the antioxidant capacity of the sprouts resulted from their interactions. As suggested by Cevallos-Casals and Cisneros-Zevallo [5], consumption of fresh sprouts could provide similar antioxidant benefits to those offered by commonly consumed fruits.

The anti-diabetic (α-amylase and α-glucosidase) and anti-obesity (pancreatic lipase) assays were used to determine biological activity of the samples (as IC_50_). Table 4 presents differences in inhibitory activity of α-amylase and α-glucosidase in the tested sprouts and microgreens. The strongest inhibition of α-amylase and α-glucosidase, constituting an alternative approach to preventing diabetes mellitus, was found in the sprouts of black medick, mung bean, beetroot, and broccoli, and in beetroot microgreens. Considerable inhibition of α-glucosidase was also determined for radish microgreens and leek sprouts. High inhibitory activity towards pancreatic lipase was noted for all investigated sprouts and microgreens (IC_50_ < 0.6), except for lentil sprouts (Table 4). Our data indicate that sprout and microgreen extracts inhibit pancreatic lipase with similar intensity and are considerably less efficient in inhibiting α-amylase and α-glucosidase. Polyphenols are well known for their capacity of binding proteins by hydrogen bonding or hydrophobic effects, leading to their complexation and precipitation. This means that extracts of different plants could potentially inhibit enzymes by aggregation.

The hypoglycemic effect of polyphenolic compounds is also due to their antioxidant potential involved in restoring the insulin-secreting machinery in pancreatic cells, or their ability to inhibit the activity of carbohydrate-hydrolyzing enzymes (α-amylase and α-glucosidase) [10,11,34].

Additionally, bioactive free amino acids are produced during germination and then in sprouts and microgreens [35]. Free amino acids, such as leucine, arginine, alanine, phenylalanine, isoleucine, lysine, and methionine, are known stimulators of insulin production and release from pancreatic cells, as they participate in the synthesis of phosphatidylinositol-3-kinase, which is an important component of insulin receptor/tyrosine kinase. Therefore, soybean sprouts may be, for example, used to reduce diabetes prevalence in Indonesia [35]. Our study confirmed a strong correlation between amino acid content and anti-diabetic (r^2^ = 0.716 and r^2^ = 0.502 for α-amylase and α-glucosidase, respectively) and anti-obesity activity (r^2^ = 0.839). For comparison, a correlation between polyphenols and anti-diabetic and anti-obesity activity was lower than r^2^ = 0.630, except for polymeric procyanidins (r^2^ > 0.940) and flavonols (r^2^ > 0.710). The beneficial health effects of sprouts of many plant species were confirmed in human studies. A randomized clinical trial [36] involving patients with type 2 diabetes showed that a four-week diet enriched in pulverized broccoli sprouts significantly lowered blood levels of triglycerides, the oxidized low density lipoprotein (LDL) to LDL ratio, the atherogenic index of plasma (API), insulin and the insulin resistance index (HOMA-IR) as compared with the control.

Inhibition of cholinesterase activity is of key importance in symptomatic treatment of Alzheimer’s disease [14]. It is crucial that the patients’ diet contain potential inhibitors that block the enzyme and thus increase the content of acetylcholine in cholinergic synapses and improve neurotransmission. Some sprouts and microgreens were characterized in terms of their capacity to inhibit the activity of acetylcholinesterase (AChE) and butylcholinesterase (BuChE), as shown in Table 4. Generally, inhibition of AChE and BuChE was species-dependent, as it was impossible to indicate which group (sprouts or microgreens) provided more effective inhibition. The strongest inhibition of AChE and BuChE was noted for radish and kale sprouts and for green bean and amaranth microgreens. Lentil and black medick sprouts and radish and beetroot microgreens were at the opposite site of the spectrum. The inhibitory effect on AChE and BuChE was stimulated by polyphenols, carotenoids and L-ascorbic acid, resulting in average correlation coefficients of r^2^ = 0.431–0.412, r^2^ = 0.386–0.387, and r^2^ = 0.577–0.507, respectively. Previous works [33,37] indicated that the inhibitory activity depended on the number and position of -OH (especially in position *orto*) and/or -OCH_3_ groups substituted in the phenyl ring. The same authors [33] reported on a strong inhibition of AChE and BuChE by chlorogenic acid and flavonol derivatives. According to Silva et al. [3], who analyzed *Glycine max* L. Merr., *Vigna radiata* L. and *Medicago sativa* L. sprout extracts, no AChE inhibitory activity was found at the tested concentrations.

### 2.7. Principal Component Analysis (PCA)

**Figure 1:**
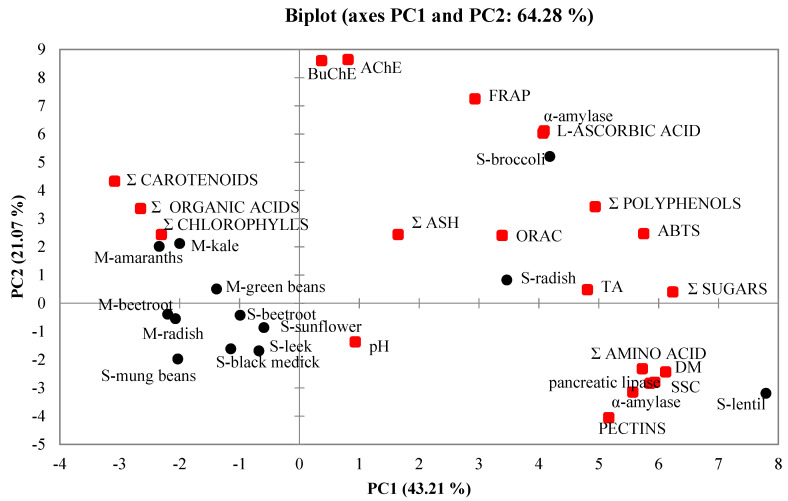
Factor plot principal component 1 (PC1) versus PC2 present the association data for sprouts and microgreens. DM—dry matter; SSC—soluble solid; TA—titratable acidity; S—sprouts; M—microgreens.

PCA was employed to better understand the relationship between biological activity and bioactive compounds and chemical composition of sprouts and microgreens. Figure 1 is a bidimensional representation of all of the variables and samples of sprouts and microgreens defined by the two first PCs as PC1 vs. PC2. Four PCs explained the cumulative percentage of total variation, while PC1 and PC2 explained 64.28% of total variation. PC1 (right side of the figure) explained 43.21% of the total variation and accounted mainly for bioactive compounds (sum of polyphenols, amino acids, and L-ascorbic acid) and biological activity (as ORAC, ABTS, FRAP, α-amylase and α-glucosidase, pancreatic lipase, AChE, and BuChE). However, antioxidant capacity might also be conferred by polyphenolic compounds and L-ascorbic acid. Broccoli, radish, and lentil sprouts were plotted among positive PC1 values, whereas the other sprout and microgreen samples were plotted in the negative half of the chart. PC2 explained 21.07% of total variation and accounted mainly for antioxidant capacity (ORAC, ABTS, FRAP), anticholinergic activity (AChE, BuChE), α-amylase, sum of polyphenols and carotenoids, chlorophylls, organic acids, L-ascorbic acid, ash, sugars, and titratable acidity. Kale, amaranth, and green pea microgreens exhibited a high total content of carotenoids and chlorophylls, contrary to lentil sprouts which were rich in amino acids. The other samples, including microgreens of beetroot and radish and sprouts of beetroot, sunflower, leek, black medick, and mung bean, were plotted mainly at the bottom of the chart due to their lowest biological activity and low content of selected bioactive compounds.

## 3. Materials and Methods 

### 3.1. Standards, Compounds and Chemicals

The following standards were used for the quantification of phenolic compounds: (−)-epicatechin; (+)-catechin; procyanidins B2, B3, C1; chlorogenic acid; and cryptochlorogenic acid from Extrasynthese (Genay, France). UPLC-grade water, prepared by using an HLP SMART 1000 s system (Hydrolab, Gdańsk, Poland), was additionally filtered through a 0.22 µm membrane filter immediately before use. Trolox (6-hydroxy-2,5,7,8-tetramethylchroman-2-carboxylic acid), phloroglucinol, hydrochloric acid, acetic acid, formic acid, sulfuric acid, ascorbic acid, acetonitrile, methanol for UPLC (gradient grade), sodium acetate, and sodium hydroxide, 3,5-dinitrosalicylic acid, potassium sodium tartrate tetrahydrate, sodium phosphate monobasic, starch from potato, *α*-amylase from porcine pancreas (type VI-8), dipotassium hydrogen orthophosphate dihydrogen, *p*-nitrophenyl-α-d-glucopyranoside, soybean lipoxygenase (type V), linoleic acid, xylenol orange and *α*-glucosidase from *Saccharomyces cerevisiae* (type I) were purchased from Sigma-Aldrich (Steinheim, Germany).

### 3.2. Plant Materials

Eight sprouts (radish (*Raphanus sativus;* Brassicaceae), lentil (*Lens culinaris;* Fabaceae), black medick (*Medicago lupulina;* Leguminosae), broccoli (*Brassica oleracea* var. italica; Brassicaceae), sunflower (*Helianthus annuus* L.; Asteraceae), leek (*Allium porrum;* Amaryllidaceae), beetroot (*Beta vulgaris;* Amaranthaceae), mung beans (*Vigna radiata;* Fabaceae); unknown variety name) and five microgreens (kale (*Brassica oleracea;* Brassicaceae), radish (*Raphanus sativus;* Brassicaceae), beetroot (*Beta vulgaris;* Amaranthaceae), green peas (*Pisum sativum;* Fabaceae), amaranth (*Amaranthus;* Amaranthaceae); unknown variety name) were obtained from a commercial shop during the period of 15–20 February 2019.

Approximately 0.5 kg of each sample was separated for 2 parts. The first part of fresh materials was used for measurements of contents of dry matter, ash, soluble solids, pH, titratable acidity, pectin, L-ascorbic acid, sugars and organic acids. The second part was freeze dried (Christ Alpha 2–4; Braun Biotech Int., Melsungen, Germany) for 24 h at a pressure of 0.220 mbar. The samples were subsequently powdered (IKA 11A basic; Staufen, Germany) for analysis, such as the content of phenolic compounds, amino acids and carotenes and then in vitro biological activity (antioxidant capacity and anti-diabetic, anti-obesity and anti-cholinesterase activity).

### 3.3. Basic Physicochemical Analyses

The dry matter (DM) expressed as % was measured using a vacuum dryer (SPT-200; ZEAMiL Horyzont; Kraków, Poland), and soluble solids (SS) expressed as °Brix content were measured using a refractometer (Atago Rx 5000; Atago Co.Ltd.; Kyoto, Japan). pH and titratable acidity (TA) expressed as g of malic acid per 100 g fresh weight (fw) were measured using an automatic pH titration system (pH- meter type IQ 150; Warsaw, Poland) as reported previously by Wojdyło et al. [38]. Total ash (%) and pectin (g/100 g fw) content were analyzed according to the method reported previously by Wojdyło et al. [29,38]. L-ascorbic acid expressed as mg per 100 g fw was determined by the HPLC-FL (Waters Co.; Milford, CT, USA) method, and sugars expressed as mg per 100 g fw were determined by HPLC-ELSD (Merck; Hitachi, Japan), while organic acid expressed as mg per 100 g fw was determined by the HPLC-PDA (Waters Co.; Milford, CT, USA) method as described previously by Wojdyło et al. [38,39]. All samples were assayed in triplicate repetition.

### 3.4. Preparation of Sprout and Microgreen Extracts for Analysis

For the determination of biological activity, the lyophilized powdered samples (approx. 1g) samples were taken, and 5 mL of methanol:water:hydrochloric acid (80:19:1, *v/v/m;* where hydrochloric acid was 37%) was added to each sample and being incubated overnight (4 °C). Following this, they were then sonicated (Sonic 6D; Polsonic, Warsaw, Poland) for 20 min [40]. Then, the extract was centrifuged (MPW-55; Warsaw, Poland) at 19,000× *g* for 10 min at 4 °C. Finally, the extract was used for the determination in vitro biological activities, such as anti-oxidant, anti-diabetic, anti-obesity, and anti-cholinergic activities. 

For the determination of polyphenolic compounds, the same protocol as that described above was used, but a methanol/water/ascorbic acid (30:68:1:1, *v/v*/m) with 1% of 37% hydrochloric acid mixture was used for extraction. Finally, before analysis, the extract was filtered by a 0.20 μm hydrophilic polytetrafluoroethylene (PTFE) membrane (Millex Simplicity Filter; Merck, Germany), and analysis by LC-PDA-Qtof-ESI-MS (identification) and by UPLC-PDA-FL (quantification) was conducted.

For the determination of carotenoid and chlorophyll [15,29] compounds, lyophilized powdered samples (aprox. 0.20 g) mixed with 10% MgCO_3_ and 1% of dibutylhydroxytoluene (BHT) were shaken (300 rpm, 30 min; DOS-10L Digital Orbital Shaker, ELMI; Riga, Latvia) with 3 mL of hexane:acetone:methanol (2:1:1, *v/v/v*) in the dark. Then, the samples were centrifuged (MPW-55; Warsaw, Poland), supernatants were collected, and residue was re-extraction additionally 4 times. Finally, all supernatants were mixed and evaporated to dryness (XcelVap^®^, Horizon Technology, Inc.; Salem, MA, USA) under nitrogen, and the residues were dissolved in methanol, filtrated by a 0.45μm nylon membrane syringe filter (VWR International, USA) and analyzed by LC-PDA-Qtof-ESI-MS (identification) and by UPLC-PDA-FL (quantification).

For the determination of amino acids [32], lyophilized powdered samples (aprox. 0.02 g) were vortexed with methanol/water (1:1, *v/v*) and sonicated (Sonic 6D, Polsonic; Warsaw, Poland) for 10 min at 20 °C. Following this, the supernatant was collected after being centrifuged (MPW-55; Warsaw, Poland) at 12,000× *g* for 10 min at 4 °C, and residue was additionally re-extracted at the same time. Supernatants were collected, and borate derivatization buffer with a pH of 8.8 was added. They were then incubated for 10 min at 55 °C with continuous vortexing using a thermo mixer (Thermomixer C, Eppendorf; Hamburg, Germany). The samples prepared were used for amino acid identification by LC-MS-PDA-Q/TOF and quantification by UPLC-PDA.

### 3.5. Estimation of Phenolic Compounds by LC-PDA-Qtof-ESI-MS (Identification) and UPLC-PDA-FL (Quantification)

Analysis of polyphenols from sprouts and microgreen leaves was carried out using an Acquity UPLC system (Waters Corp., Milford, MA, USA) equipped with a photodiode (PDA) and fluorescence (FL) detector with a binary solvent manager (Waters Corp., Milford, MA, USA) series and with a mass detector G2 Qtof mass spectrometer (Waters, Manchester, UK) equipped with an electrospray ionization (ESI) source operating in negative modes. For polyphenolic identification and quantification, 5 μL of each sample was analyzed using a BEH C18 column (2.1 × 100 mm, 1.7 μm; Waters Corp.; Dublin, Ireland) at 30 °C with gradient elution at a flow rate of 0.42 mL/min for a duration of 15 min. The mobile phase was composed of solvent A (2.0% formic acid) and solvent B (acetonitrile), when B at 1% to 25% until 12 min; then, it was held constant to wash and re-equilibrate the column. The remaining parameters for LC-PDA-Qtof-ESI-MS were as follows: scanning from *m/z* 100 to 1200; capillary voltage of 2000 V; cone voltage of 35 V; source and desolvation temperature of 100 and 250 °C, respectively; and desolvation gas (nitrogen) flow rate of 300 L/h. The characterization of the single components was carried out via the retention time and the accurate molecular masses at negative and positive ion mode, and it was set to the base peak intensity (BPI) chromatograms. The data were collected by MassLynxTM 4.1 ChromaLynx Application Manager (Waters Corp., Milford, MA, USA) software.

Retention times (Rt) and spectra (λ) were compared with those of pure standards. Quantification was achieved by injection of solutions of known concentrations ranging from 0.05 to 0.5 mg/mL (R^2^ ≤ 0.9998) made from (−)-epicatechin, (+)-catechin, and procyanidin B1 for flavan-3-ols at 280 nm; chlorogenic, neochlorogenic and 3,5-di-caffeoylquinic acids for phenolic acid at 320 nm; quercetin- and kaempferol-3-*O*-glucoside, isorhamnetin-3-*O*-glucoside and genistein for flavonols and isoflavonols at 360 nm; and cyanidin-3-*O*-glucoside for anthocyanins at 520 nm. All samples were assayed in triplicate, and the results were expressed as mg per kg of dry weight (dw).

Analysis of polymeric procyanidins was performed by the phloroglucinolysis method according to the method of Wojdyło et al. [40]. Samples (aprox. 0.05 g) of powder were put into 2 mL Eppendorf vials; then, 0.8 and 0.4 mL of methanolic solution of phloroglucinol and methanolic HCl, respectively, were added, and the samples were incubated for 30 min at 50 °C with continuous vortexing using a thermo mixer (Thermomixer C, Eppendorf; Germany). After reaction, 0.6 mL of sodium acetate buffer was added and cooled in ice water; it was then centrifuged immediately at 12,000× *g* for 10 min at 4 °C (MPW-55; Warsaw, Poland). The analysis of polymeric procyanidins was carried out on a UPLC-FL Acquity system (Waters Corp., Waters Corp.; Ireland), and detection was recorded at an emission wavelength of 360 nm and an excitation wavelength of 278 nm. An injection of 5 μL of each sample was analyzed using a BEH C18 RP column (2.1 × 5 mm, 1.7 μm; Waters Corporation, Milford, MA, USA) at 15 °C with gradient elution at a flow rate of 0.42 mL/min for a duration of 10 min. The mobile phase was composed of solvent A (2.5% acetic acid) and solvent B (acetonitrile), when 2% B initially until 0.6 min followed by 9% B until 7.3 min; then this was held constant to wash and re-equilibrate the column until 10 min. The calibration curves, which were based on the peak area, were established using (+)-catechin, (−)-epicatechin, and procyanidin B1 after the phloroglucinol reaction as (+)-catechin− and (−)-epicatechin−phloroglucinol adduct standards. All samples were assayed in triplicate, and the results were expressed as mg per kg of dry weight (dw).

### 3.6. Estimation of Carotenoids and Chlorophylls by LC-PDA-Qtof-ESI-MS (Identification) and UPLC-PDA (Quantification)

Estimation of carotenoids from sprouts and microgreen leaves was carried out using an Acquity system as polyphenol compounds as described previously by Wojdyło et al. [29] and Tkacz et al. [15]. For carotenoid identification and quantification, 10 μL of each sample was analyzed using a BEH RP C18 column (2.1 × 100 mm, 1.7 μm; Waters Corp.; Ireland) at 32 °C with a gradient elution at a flow rate of 0.5 mL/min for a duration of 16.60 min. The mobile phase was composed of solvent A (0.1% formic acid) and solvent B (acetonitrile:methanol, 7:3, *v/v*), when 25% of A until 0.6 min, 4.9% of A until 6.5 min, and 0% of A until 13.6 min; then, that was held constant to wash and re-equilibrate the column. The remaining parameters for the analysis of carotenoid and chlorophyll compounds by LC-PDA-Qtof-ESI-MS and UPLC-PDA were the same as those for phenolic compounds except for a desolvation gas (nitrogen) flow rate of 350 L/h. The characterization of the single components was carried out via the retention time and the accurate molecular masses at positive ion mode, and it was set to the base peak intensity (BPI) chromatograms. Retention times (Rt) and spectra (λ) were compared with those of pure standards. Quantification was achieved by injection of solutions of known concentrations ranging from 0.05 to 0.5 mg/mL (R^2^ ≤ 0.9997) made from carotenoids (lutein, zeaxanthin, and ß-carotene) at 450 nm and for chlorophylls (chlorophylls *a*, pheophytin *a*) at 650 nm. All samples were assayed in triplicate, and the results were expressed as mg per kg of dry weight (dw).

### 3.7. Estimation of Amino Acid by LC-PDA-Qtof-ESI-MS (Identification) and UPLC-PDA (Quantification)

Estimation of amino acids from sprouts and microgreen leaves was carried out using an Acquity system as polyphenol compounds as described previously by Riga et al. [20] and Collado-González et al. [32]. For amino acid identification and quantification, 3 μL of each sample was analyzed using an AccQ Tag Ultra BEH column (2.1 × 100 mm, 1.7 μm; Waters Corp.; Ireland) at 50 °C with a gradient elution at a flow rate of 0.5 mL/min for a duration of 15 min. The mobile phase was composed of solvent A (mix of acetonitrile:formic acid:ammonium acetate (10:6:84, *v/v/v*) and solvent B (acetonitrile:formic acid, 99.9:0.1, *v/v*) where 99% of A until 0.3 min, 97% of A until 3.2 min, and 40% of A until 11.0 min; then, it was held constant to wash and re-equilibrate the column. The remaining parameters for the analysis of amino acid compounds by LC-PDA-Qtof-ESI-MS and UPLC-PDA were the same as phenolic compounds, except for the scanning mode from *m/z* 100 to 700, the capillary voltage of 2500 V, the cone voltage of 30 V, the desolvation temperature of 350 °C, and the desolvation gas (nitrogen) flow rate of 535 L/h. The characterization of the single components was carried out via the retention time and the accurate molecular masses at negative ion mode, and it was set to the base peak intensity (BPI) chromatograms. Retention times (Rt) and spectra (λ) were compared with those of pure standards. Quantification was achieved by injection of solutions of known concentrations ranging from 20 to 100 mg/L (R^2^ ≤ 0.9998) made from amino acid at 260 nm. All samples were assayed in triplicate, and the results were expressed as mg per 100 g of fw.

### 3.8. Analysis of Biological Activity

*Anti-oxidant capacity*. The oxygen radical absorbance capacity (ORAC), ferric reducing ability of plasma (FRAP) and 2,2′-azino-bis(3-ethylbenzothiazoline-6-sulphonic acid) (ABTS) antioxidant assays were determined as previously described by Ou et al. [41], Benzie and Strain [42], and Re et al. [43], respectively. The results were expressed as mM Trolox per 100 g of dm.

*Anti-diabetic and anti-obesity activity*. The α-amylase, α-glucosidase and pancreatic lipase were determined as previously described by Nowicka and Wojdyło [34] and Tkacz et al. [15]. The acarbose and orlistat was included as a positive control for α-amylase, α-glucosidase and pancreatic lipase, respectively, and the obtained results are presented as IC50 in mg/mL, i.e., the amount of the sample that is able to reduce enzyme activity by 50%.

*Anti-cholinergic activity.* The acetylcholinesterase (AChE) and butylcholinesterase (BuChE) inhibitions were determined as previously described by Wojdyło et al. [37]. The results are expressed as % of inhibition.

All tests: anti-oxidant (ABTS, ORAC, FRAP), α-amylase, α-glucosidase, anti-lipase, and anti-cholinergic activity were performed in triplicate using a microplate reader SynergyTM H1 (BioTek, Winooski, VT, USA).

### 3.9. Statistical Analysis

Statistical analysis was conducted using *XLSTAT* 2017 (Addinsoft, New York, NY, USA). Statistically significant differences at *p* ≤ 0.05 between all means were analysis by one-way analysis of variance (ANOVA) by Tukey’s multiple range test. Principal components analysis (PCA) was performed to highlight correlations. All samples were assayed in triplicate and presented in tables as mean ± standard deviation.

## 4. Conclusions

Given the current interest in edible plants, this study notably improved our knowledge on metabolic profile of sprouts and microgreens of dietary species, revealing that they are good sources of bioactive compounds with health-promoting properties. The results of this work show that sprouts have strong antioxidant capacity due to high contents of polyphenols and L-ascorbic acid. Sprouts are also a better source of amino acids, pectins and sugars than microgreens. Microgreens contain high levels of carotenoids, chlorophylls and organic acids but scarce amounts of sugars. They also show higher anti-diabetic and anti-cholinergic activity than sprouts. Selected sprouts (broccoli, radish, lentil) and microgreens (radish, amaranths, kale) should be used daily as superfoods or functional food. Consumption of sprouts and microgreens can be of magnificent importance for humans to stay healthy and avoid civilization diseases associated with oxidative stress.

## Figures and Tables

**Table 1:** Basic chemical composition (dry matter, pH, titratable acidity, pectins, ash), L-ascorbic acid, sugars and organic acids of sprouts and microgreens.

Compounds	Sprouts								Microgreens				
	Radish (*Raphanus sativus*)	Lentil (*Lens culinaris*)	Black Medick (*Medicago lupulina*)	Broccoli (*Brassica oleracea* var. Italica)	Sunflower (*Helianthus annuus* L.)	Leek (*Allium porrum*)	Beetroot (*Beta vulgaris*)	Mung Beans (*Vigna radiata*)	Kale (*Brassica oleracea*)	Radish (*Raphanus sativus*)	Beetroot (*Beta vulgaris*)	Green Peas *(Pisum sativum)*	Amaranths (*Amaranthus*)
Dry matter	16.5 ± 0.1 ^‡^b^†^	46.4 ± 0.2a	6.2 ± 0.0g	15.8 ± 0.4c	8.0 ± 0.5e	8.6 ± 0.1d	6.9 ± 0.6 f	4.7 ± 0.2i	4.1 ± 0.2j	5.0 ± 0.3i	5.5 ± 0.4h	8.1 ± 0.1e	5.4 ± 0.1h
SSC	11.5 ± 0.2b	33.2 ± 0.2a	4.2 ± 0.1g	9.0 ± 0.1c	4.8 ± 0.9f	5.5 ± 0.4e	3.4 ± 0.5hi	3.4 ± 0.2hi	3.1 ± 0.3i	3.2 ± 0.4i	3.5 ± 0.2hi	6.3 ± 0.3d	3.8 ± 0.2gh
pH	5.3 ± 0.1i	6.6 ± 0.0a	5.6 ± 0.1g	5.5 ± 0.3h	5.5 ± 0.4h	5.6 ± 0.2g	6.4 ± 0.5b	5.3 ± 0.3i	6.2 ± 0.4c	5.7 ± 0.2f	6.1 ± 0.3d	6.0 ± 0.4e	6.0 ± 0.3e
TA	0.7 ± 0.1a	0.5 ± 0.0b	0.4 ± 0.0c	0.5 ± 0.0b	0.2 ± 0.1gh	0.4 ± 0.1c	0.2 ± 0.0fg	0.3 ± 0.0d	0.2 ± 0.0h	0.3 ± 0.2e	0.2 ± 0.0f	0.4 ± 0.1c	0.2 ± 0.0f
Pectins	0.3 ± 0.0b	3.1 ± 0.0a	0.0 ± 0.0c	0.0 ± 0.0c	0.0 ± 0.0c	0.0 ± 0.0c	0.0 ± 0.0c	0.0 ± 0.0c	0.0 ± 0.0c	0.0 ± 0.0c	0.0 ± 0.0c	0.0 ± 0.0c	0.0 ± 0.0c
Σ Ash	0.8 ± 0.1e	1.3 ± 0.0b	0.4 ± 0.1i	0.7 ± 0.0e	0.3 ± 0.0i	0.5 ± 0.2g	0.7 ± 0.0f	0.2 ± 0.3j	1.1 ± 0.1c	0.7 ± 0.0f	0.9 ± 0.4d	0.4 ± 0.0h	1.6 ± 0.2a
L-ascorbic acid	93.4 ± 2.0b	28.3 ± 1.0d	11.0 ± 0.8g	114.0 ± 2.7a	9.7 ± 0.3g	9.1 ± 0.7gh	2.8 ± 0.1j	7.1 ± 0.2hi	20.3 ± 0.7f	24.7 ± 1.2e	6.6 ± 0.3i	31.1 ± 1.4c	8.9 ± 0.8gh
Sugars (g/100 g fw)													
Glucose	0.9 ± 0.1ab	0.1 ± 0.0c	0.0 ± 0.0d	1.0 ± 0.1a	0.0 ± 0.0d	0.0 ± 0.0d	0.0 ± 0.0d	0.0 ± 0.0d	0.0 ± 0.0d	0.0 ± 0.0d	0.0 ± 0.0d	0.0 ± 0.0d	0.0 ± 0.0d
Fructose	0.3 ± 0.1b	0.2 ± 0.0c	0.1 ± 0.0d	0.5 ± 0.0a	0.2 ± 0.0c	0.4 ± 0.1a	0.0 ± 0.0e	0.2 ± 0.0c	0.0 ± 0.0e	0.0 ± 0.0e	0.0 ± 0.0e	0.0 ± 0.0e	0.0 ± 0.0e
Saccharose	0.0 ± 0.0c	1.4 ± 0.1a	0.1 ± 0.0b	0.0 ± 0.0c	0.0 ± 0.0c	0.0 ± 0.0c	0.0 ± 0.0c	0.0 ± 0.0c	0.0 ± 0.0c	0.0 ± 0.0c	0.0 ± 0.0c	0.0 ± 0.0c	0.0 ± 0.0c
Sorbitol	0.0 ± 0.0c	0.0 ± 0.0c	0.0 ± 0.0c	0.0 ± 0.0c	0.6 ± 0.1a	0.1 ± 0.0b	0.0 ± 0.0a	0.0 ± 0.0c	0.0 ± 0.0c	0.0 ± 0.0c	0.0 ± 0.0c	0.0 ± 0.0c	0.0 ± 0.0c
Mannose	0.0 ± 0.0b	0.0 ± 0.0b	0.0 ± 0.0b	0.0 ± 0.0b	0.1 ± 0.0a	0.0 ± 0.0b	0.0 ± 0.0b	0.0 ± 0.0b	0.0 ± 0.0b	0.0 ± 0.0b	0.0 ± 0.0b	0.0 ± 0.0b	0.0 ± 0.0b
Rhamnose	0.0 ± 0.0b	0.1 ± 0.00a	0.0 ± 0.0b	0.0 ± 0.0b	0.0 ± 0.0b	0.0 ± 0.0b	0.0 ± 0.0b	0.0 ± 0.0b	0.0 ± 0.0b	0.0 ± 0.0b	0.0 ± 0.0b	0.0 ± 0.0b	0.0 ± 0.0b
Σ Sugars	1.2c	1.8a	0.2f	1.5b	0.9d	0.5e	0.0 ± 0.0g	0.2f	0.0 ± 0.0g	0.0 ± 0.0g	0.0 ± 0.0g	0.0 ± 0.0g	0.0 ± 0.0g
Organic acids (g/100 g fw)													
Phytic acid	0.0 ± 0.0d	0.0 ± 0.0d	0.1 ± 0.0c	0.0 ± 0.0d	0.7 ± 0.1b	0.0 ± 0.0d	2.8 ± 0.1a	0.0 ± 0.0d	0.0 ± 0.0d	0.0 ± 0.0d	0.0 ± 0.0d	0.0 ± 0.0d	0.0 ± 0.0d
Oxalic acid	1.4 ± 0.2f	1.5 ± 0.2f	0.5 ± 0.0g	1.9 ± 0.3e	0.5 ± 0.1g	2.2 ± 0.3e	2.3 ± 0.3e	0.7 ± 0.1g	98.8 ± 1.6b	79.3 ± 1.3c	13.4 ± 0.3d	0.3 ± 0.0g	120.8 ± 1.3a
Citric acid	0.1 ± 0.0c	0.5 ± 0.1b	0.2 ± 0.0c	0.2 ± 0.0c	0.1 ± 0.0c	1.6 ± 0.2a	0.2 ± 0.0c	0.1 ± 0.0c	0.0 ± 0.0d	0.0 ± 0.0d	0.0 ± 0.0d	0.5 ± 0.0b	0.0 ± 0.0d
Malic acid	0.2 ± 0.0c	0.3 ± 0.0b	0.4 ± 0.1a	0.3 ± 0.0b	0.1 ± 0.0d	0.2 ± 0.0c	0.1 ± 0.0d	0.0 ± 0.0e	0.0 ± 0.0e	0.0 ± 0.0e	0.0 ± 0.0e	0.0 ± 0.0e	0.0 ± 0.0e
Quinic acid	0.1 ± 0.0c	0.0 ± 0.0d	0.3 ± 0.0b	0.1 ± 0.0c	0.4 ± 0.0a	0.0 ± 0.0d	0.0 ± 0.0d	0.1 ± 0.0c	0.0 ± 0.0d	0.0 ± 0.0d	0.0 ± 0.0d	0.0 ± 0.0d	0.0 ± 0.0d
Succinic	0.5 ± 0.0c	0.8 ± 0.1ab	0.0 ± 0.0e	1.0 ± 0.1a	0.4 ± 0.0c	0.4 ± 0.0c	0.0 ± 0.0e	0.0 ± 0.0e	0.0 ± 0.0e	0.0 ± 0.0e	0.0 ± 0.0e	0.2 ± 0.0d	0.0 ± 0.0e
Other organic acid	0.5 ± 0.1c	0.2 ± 0.0e	0.6 ± 0.1c	0.4 ± 0.0d	0.1 ± 0.0e	0.2 ± 0.0e	0.0 ± 0.0f	1.7 ± 0.2ab	0.0 ± 0.0f	0.0 ± 0.0f	0.0 ± 0.0f	2.0 ± 0.1a	0.0 ± 0.0f
Σ Organic acids	2.6e	3.2e	2.2e	3.9e	2.3e	4.5e	5.4e	2.6e	98.8b	79.3c	13.4d	3.0e	120.8a
ANOVA test ^†^		Dry matter		Soluble solid	pH	Titratable acidity		L-ascorbic acid	Σ Ash	Pectins	Σ Sugars	Σ Organic acids	
Sprouts		A		A	B	A		A	B	A	A	B	
Microgreens		B		B	A	B		B	A	B	B	A	

nd—not detected; dm—dry matter [%]; SSC—soluble solid (^o^Bx); TA—titratable acidity (g malic acid/100 g fw); pectins (%); ash (%); L-ascorbic acid (mg/100 g fw); ^†^ significant at *p* < 0.05; ^‡^ values (mean of three replications) followed by the same letter within the same column were not significantly different (*p* < 0.05) according to Tukey’s least significant difference test.

**Table 2:** Polyphenols [mg/100 g fw], carotenoids and chlorophylls [μg/g fw] of sprouts and microgreens.

Compounds	Sprouts								Microgreens				
	Radish (*Raphanus sativus*)	Lentil (*Lens culinaris*)	Black Medick (*Medicago lupulina*)	Broccoli (*Brassica oleracea* var. Italica)	Sunflower (*Helianthus annuus* L.)	Leek (*Allium porrum*)	Beetroot (*Beta vulgaris*)	Mung Beans (*Vigna radiata*)	Kale (*Brassica oleracea*)	Radish (*Raphanus sativus*)	Beetroot (*Beta vulgaris*)	Green Peas *(Pisum sativum)*	Amaranths (*Amaranthus)*
*Polyphenolic compounds*													
Flavan-3-ols	40.3 ± 3.9b ^‡^	34.9 ± 2.7c	29.9 ± 1.8c	53.9 ± 1.1a	24.9 ± 0.5d	18.3 ± 0.6e	24.7 ± 1.2d	22.1 ± 0.9d	21.2 ± 0.7d	1.9 ± 0.1g	9.8 ± 0.5f	27.6 ± 2.4c	8.8 ± 0.5f
Polymeric procyanidins	39.8 ± 1.5b	94.5 ± 3.1a	6.3 ± 0.3f	26.6 ± 1.3c	2.1 ± 0.1i	6.7 ± 0.1g	3.3 ± 0.3g	1.4 ± 0.3i	8.3 ± 0.2e	3.6 ± 0.2g	7.3 ± 0.7ef	14.4 ± 1.2d	1.3 ± 0.1i
Phenolic acid	100.0 ± 2.5c	7.9 ± 0.3g	6.4 ± 0.4g	110.2 ± 1.4b	125.6 ± 2.5a	6.5 ± 0.2g	5.8 ± 0.7g	2.7 ± 0.2h	15.1 ± 0.1f	1.5 ± 0.4h	16.6 ± 2.1f	41.9 ± 3.3e	58.6 ± 1.6d
Flavonols + flavones	1.2 ± 0.0d	2.0 ± 0.2c	0.0 ± 0.0f	0.0 ± 0.0f	0.0 ± 0.0f	0.0 ± 0.0f	1.8 ± 0.1c	0.5 ± 0.0e	0.0 ± 0.0f	0.0 ± 0.0f	6.9 ± 0.5a	4.6 ± 0.6b	0.3 ± 0.0e
Isoflavones	0.0 ± 0.0c	40.5 ± 2.4a	0.0 ± 0.0c	0.0 ± 0.0c	0.0 ± 0.0c	0.0 ± 0.0c	0.0 ± 0.0c	0.0 ± 0.0c	0.0 ± 0.0c	0.0 ± 0.0c	0.0 ± 0.0c	20.0 ± 0.8b	0.0 ± 0.0c
Anthocyanins	2.1 ± 0.1d	0.0 ± 0.0f	0.1 ± 0.0e	0.5 ± 0.0e	0.0 ± 0.0f	0.0 ± 0.0f	2.3 ± 0.4d	0.0 ± 0.0f	0.0 ± 0.0f	17.2 ± 0.3b	5.5 ± 0.3c	0.0 ± 0.0f	63.9 ± 2.3a
*Σ Polyphenols*	183.4b	179.8b	42.8f	191.1a	152.6c	31.5hi	35.6gh	26.7ij	44.7f	24.3j	40.7f	108.5e	132.9de
ANOVA test ^†^	A								B				
*Chlorophylls*													
Chlorophylls *b*	3.5 ± 0.1i	16.1 ± 0.6f	1.6 ± 0.1g	2.7 ± 0.4	13.8 ± 0.3g	5.9 ± 0.2h	2.6 ± 0.3j	1.0 ± 0.2h	57.0 ± 1.2e	64.7 ± 1.5d	68.3 ± 1.4c	157.8 ± 2.4b	186.3 ± 2.1a
Pheophytin *b*	0.0 ± 0.0c	0.0 ± 0.0c	0.0 ± 0.0c	0.0 ± 0.0c	0.0 ± 0.0c	0.0 ± 0.0c	0.0 ± 0.0c	0.0 ± 0.0c	6.4 ± 0.3a	0.0 ± 0.0c	5.7 ± 0.2b	0.0 ± 0.0c	0.0 ± 0.0c
chlorophylls *b’*	0.0 ± 0.0h	0.0 ± 0.0h	0.0 ± 0.0h	0.0 ± 0.0h	2.4 ± 0.2e	1.2 ± 0.3f	0.4 ± 0.1g	0.0 ± 0.0h	10.9 ± 0.7c	7.5 ± 0.8d	9.7 ± 0.2c	14.8 ± 1.4b	17.3 ± 0.7a
Pheophytin *b’*	0.0 ± 0.0d	0.0 ± 0.0d	0.0 ± 0.0d	0.0 ± 0.0d	0.0 ± 0.0d	0.0 ± 0.0d	0.0 ± 0.0d	0.0 ± 0.0d	1.9 ± 0.1b	0.0 ± 0.0d	0.9 ± 0.1c	3.1 ± 0.3a	2.1 ± 0.2b
Chlorophylls *a*	27.9 ± 1.1f	77.6 ± 1.5e	10.5 ± 0.7h	11.0 ± 0.6h	29.9 ± 1.1f	7.7 ± 0.2i	23.0 ± 0.5g	3.9 ± 0.1j	88.1 ± 1.4d	121.2 ± 2.1c	125.6 ± 2.4c	288.3 ± 3.6b	336.2 ± 3.2a
Chlorophylls *a’*	3.5 ± 0.2g	7.6 ± 0.3e	1.3 ± 0.3h	1.0 ± 0.1i	3.1 ± 0.1g	4.7 ± 0.4f	1.8 ± 0.2h	0.0 ± 0.0j	12.7 ± 1.2c	6.4 ± 0.3e	9.5 ± 0.1d	20.3 ± 1.5a	16.9 ± 1.1b
Pheophytin *a*	2.3 ± 0.3f	7.2 ± 0.7d	1.0 ± 0.1h	1.7 ± 0.0g	3.0 ± 0.2f	5.5 ± 0.2e	2.7 ± 0.1f	1.1 ± 0.3h	16.0 ± 0.7c	14.7 ± 0.7c	35.2 ± 0.5b	35.3 ± 01b	75.4 ± 0.3a
Pheophytin *a’*	0.0 ± 0.0e	0.0 ± 0.0e	0.0 ± 0.0e	0.0 ± 0.0e	0.0 ± 0.0e	1.5 ± 0.5d	0.0 ± 0.0e	0.0 ± 0.0e	2.6 ± 0.2c	1.2 ± 0.1d	3.8 ± 0.5b	3.2 ± 0.1c	4.3 ± 0.2a
*Σ Chlorophylls*	37.2fg	108.5e	14.3hi	16.4ghi	52.2f	26.5ghi	30.5fgh	6.0i	195.6d	215.7d	258.7c	522.7b	638.5a
ANOVA test^†^	B								A				
*Carotenoids*													
Neochrome	21.0 ± 0.4g	0.0 ± 0.0l	5.4 ± 0.1j	10.6 ± 1.0i	29.1 ± 1.2f	18.3 ± 1.1g	15.7 ± 1.3h	1.2 ± 0.3k	107.5 ± 1.3c	99.9 ± 1.1d	86.4 ± 1.2e	255.0 ± 2.9b	301.3 ± 4.0a
Neoxanthin	98.5 ± 1.6c	2.7 ± 0.3h	45.7 ± 1.7e	56.4 ± 2.7d	32.4 ± 2.1f	18.4 ± 0.9g	55.2 ± 2.5d	3.0 ± 0.2h	3.5 ± 0.5h	90.5 ± 2.6c	2.8 ± 1.1h	256.8 ± 4.1b	293.8 ± 1.3a
Zeoxanthin	38.1 ± 1.1d	0.0 ± 0.0j	6.7 ± 0.2g	23.5 ± 1.8e	20.2 ± 1.4e	21.7 ± 1.3e	36.9 ± 1.1d	1.1 ± 0.1i	3.2 ± 0.2h	42.7 ± 1.4c	17.4 ± 0.3f	57.1 ± 2.8b	132.2 ± 2.6a
Lutein	570.6 ± 2.7d	31.0 ± 1.4j	113.2 ± 2.1i	193.2 ± 3.6g	382.0 ± 3.5e	168.6 ± 2.5h	305.2 ± 3.8f	13.2 ± 0.7k	629.6 ± 41c	565.2 ± 1.8d	684.4 ± 4.2b	1435.7 ± 2.6a	1478.9 ± 1.2a
Violaxanthin	37.9 ± 1.1a	0.0 ± 0.0g	4.3 ± 0.1e	37.2 ± 1.2a	14.0 ± 1.0d	21.5 ± 0.7b	12.1 ± 1.3d	0.5 ± 0.0f	35.8 ± 1.1a	8.3 ± 2.5e	37.0 ± 1.1a	16.9 ± 1.1c	36.6 ± 1.9a
(*α+β*)-Carotene	41.7 ± 0.5g	0.0 ± 0.0j	10.1 ± 0.2i	23.3 ± 2.6i	26.4 ± 1.0i	31.7 ± 1.0h	139.8 ± 1.8f	0.9 ± 0.1k	2239.0 ± 5.9a	699.9 ± 4.8e	1249.1 ± 5.2c	728.4 ± 4.2d	1769.2 ± 5.2b
other carotenoids	141.0 ± 1.1a	2.8 ± 0.3g	12.1 ± 1.3f	107.6 ± 3.6b	25.4 ± 1.0e	60.9 ± 2.1c	41.0 ± 1.1d	2.5 ± 0.2g	68.5 ± 1.7c	3.7 ± 0.2g	22.8 ± 1.1e	44.4 ± 0.5d	61.6 ± 1.1c
*Σ Carotenoids*	948.8f	36.4j	197.5i	451.7gh	529.5g	341.0hi	605.8g	22.5j	3087.1b	1510.1e	2099.9d	2794.4c	4073.5a
ANOVA test ^†^	B								A				

^†^ Significant at *p* < 0.05; ^‡^ values (mean of three replications) followed by the same letter within the same column were not significantly different (*p* < 0.05) according to Tukey’s least significant difference test.

**Table 3:** Free amino acid (mg/100 g fw) composition of sprouts and microgreens.

Amino acid	Sprouts								Microgreens				
	Radish (*Raphanus sativus*)	Lentil (*Lens culinaris*)	Black Medick (*Medicago lupulina*)	Broccoli (*Brassica oleracea* var. Italica)	Sunflower (*Helianthus annuus* L.)	Leek (*Allium porrum*)	Beetroot (*Beta vulgaris*)	Mung Beans (*Vigna radiata*)	Kale (*Brassica oleracea*)	Radish (*Raphanus sativus*)	Beetroot (*Beta vulgaris*)	Green Peas *(Pisum sativum)*	Amaranths (*Amaranthus)*
L-Histidine	43.3 ± 2.1b	92.6 ± 2.4a	20.9 ± 1.3c	41.5 ± 3.2b	13.8 ± 1.5e	16.9 ± 1.1d	12.8 ± 1.4e	22.8 ± 2.1c	10.6 ± 0.9e	13.1 ± 1.1e	2.3 ± 0.3g	7.7 ± 1.1f	1.5 ± 0.2g
L-Asparagine	30.3 ± 2.8i	438.5 ± 4.2a	317.8 ± 8.6b	46.3 ± 1.3h	56.1 ± 2.4g	181.8 ± 5.2d	16.2 ± 0.3j	211.7 ± 7.3c	69.7 ± 3.6f	221.4 ± 5.2c	4.3 ± 0.1l	106.2 ± 4.1e	6.7 ± 0.3k
L-Arginine	10.0 ± 1.0g	36.1 ± 1.2d	39.8 ± 2.3d	39.8 ± 2.2d	70.2 ± 1.2b	53.6 ± 1.3c	55.9 ± 2.5c	52.5 ± 4.1c	32.3 ± 1.3d	18.2 ± 1.1f	28.3 ± 0.4e	83.2 ± 2.5a	27.5 ± 1.1e
L-Serine	30.2 ± 2.1b	25.9 ± 1.2c	8.7 ± 1.1f	36.8 ± 1.0ab	39.5 ± 3.1a	29.2 ± 6.3b	22.9 ± 1.1c	16.0 ± 1.2d	23.2 ± 1.1c	10.7 ± 0.5e	4.9 ± 0.2	12.9 ± 1.0e	7.6 ± 0.4f
L-Glutamine	203.3 ± 4.9c	73.7 ± 3.2f	32.1 ± 4.2h	330.6 ± 5.7a	145.5 ± 5.3d	260.1 ± 7.3b	90.6 ± 4.2e	11.4 ± 1.7i	98.8 ± 3.8e	4.9 ± 0.2j	25.7 ± 0.5h	53.5 ± 2.6g	48.0 ± 1.1g
L-Glycine	18.8 ± 3.1b	20.8 ± 1.1b	5.4 ± 0.5e	20.0 ± 1.1b	10.2 ± 1.1c	12.3 ± 1.1c	7.7 ± 0.2d	3.3 ± 0.5f	1.3 ± 0.2g	35.4 ± 1.5a	3.9 ± 0.1f	10.3 ± 1.0c	4.0 ± 0.2f
L-Aspartic acid	17.1 ± 1.1f	151.4 ± 2.5a	12.3 ± 1.4g	20.0 ± 0.7e	11.7 ± 0.6g	23.0 ± 2.1e	32.5 ± 1.1c	6.6 ± 0.6h	13.3 ± 0.1g	25.2 ± 2.1d	48.0 ± 2.1b	27.0 ± 1.0d	36.1 ± 1.2c
L-Glutamic acid	21.1 ± 2.4b	77.2 ± 3.2a	18.3 ± 1.3b	20.8 ± 0.5b	4.7 ± 0.2f	23.4 ± 1.2b	6.9 ± 0.4e	2.0 ± 0.2g	13.5 ± 0.5c	16.7 ± 1.0c	12.3 ± 1.9d	16.6 ± 2.10c	14.8 ± 0.5c
L-Threonine	27.3 ± 2.3b	40.6 ± 2.0a	20.7 ± 1.0c	40.5 ± 1.2a	12.3 ± 0.9d	21.0 ± 2.0c	5.0 ± 0.7e	13.8 ± 0.9d	4.8 ± 0.2e	4.4 ± 0.5e	1.7 ± 0.2g	5.8 ± 1.1e	2.9 ± 0.0f
L-Alanine	14.5 ± 1.3e	47.5 ± 1.1a	16.8 ± 0.6d	41.3 ± 2.1ab	17.4 ± 1.1d	21.5 ± 1.6c	9.7 ± 1.3f	7.5 ± 0.4f	4.6 ± 0.1g	6.7 ± 0.7f	6.2 ± 0.1f	6.9 ± 1.0f	10.0 ± 0.1f
γ-Amino n-butyric acid	7.3 ± 0.9c	46.3 ± 0.8a	5.3 ± 0.2e	10.3 ± 1.1b	9.2 ± 0.5b	6.4 ± 0.4d	6.2 ± 0.4d	nd	0.5 ± 0.0h	8.7 ± 0.4c	1.6 ± 1.1g	3.4 ± 2.1f	5.7 ± 0.5e
L-Ornithine	29.2 ± 2.1a	nd	nd	nd	nd	nd	nd	nd	2.4 ± 0.2b	nd	nd	nd	nd
L-Proline	29.9 ± 1.5b	113.2 ± 4.6a	5.4 ± 0.7d	28.7 ± 1.4b	13.6 ± 1.3c	3.6 ± 0.1e	nd	10.6 ± 0.8c	1.5 ± 0.4f	1.4 ± 0.0f	0.7 ± 0.1g	1.8 ± 1.1f	1.5 ± 0.3f
L-Cystine	1.7 ± 0.2f	nd	1.0 ± 0.1g	2.7 ± 0.3e	16.8 ± 0.4a	4.6 ± 0.4d	13.4 ± 1.3b	nd	0.7 ± 0.1g	17.4 ± 0.7a	0.3 ± 0.0h	8.5 ± 0.3c	5.8 ± 0.1d
L-Lysine	15.8 ± 1.2c	14.7 ± 1.2c	5.5 ± 0.3e	27.7 ± 0.7a	8.5 ± 0.5d	17.5 ± 1.3b	7.1 ± 0.4d	14.2 ± 1.1c	4.3 ± 0.2e	8.4 ± 0.3d	3.8 ± 0.4e	13.5 ± 0.9c	3.5 ± 0.3e
L-Tyrosine	10.1 ± 0.7d	10.4 ± 2.1d	3.5 ± 0.2f	19.3 ± 1.4a	5.1 ± 0.4e	13.6 ± 2.1c	3.1 ± 0.2f	16.7 ± 1.6b	2.6 ± 0.1g	4.6 ± 0.1e	1.6 ± 0.1h	3.2 ± 0.1f	3.7 ± 0.1f
L-Methionine	1.3 ± 0.2c	1.5 ± 1.1b	0.8 ± 0.1e	1.2 ± 0.1cd	1.5 ± 1.1b	1.1 ± 0.1d	nd	3.9 ± 0.4a	1.6 ± 0.1b	0.9 ± 0.0e	nd	1.0 ± 0.2d	nd
L-Valine	28.6 ± 0.5c	45.9 ± 3.2a	21.6 ± 1.1d	47.1 ± 3.2a	15.7 ± 2.1e	9.8 ± 0.3f	7.7 ± 0.6g	34.6 ± 2.7b	14.5 ± 0.4e	19.1 ± 0.7d	4.3 ± 0.4h	11.7 ± 0.2f	4.5 ± 0.4h
L-Homocysteine	nd	nd	1.5 ± 0.1a	nd	nd	nd	nd	nd	0.2 ± 0.0b	nd	nd	nd	nd
L-Isoleucine	18.4 ± 0.2b	12.0 ± 1.1d	9.6 ± 0.6e	30.8 ± 2.1a	14.7 ± 1.6c	7.3 ± 0.4f	7.5 ± 0.9f	28.0 ± 3.1a	5.4 ± 0.3g	8.9 ± 0.3e	2.5 ± 0.1h	5.0 ± 0.1g	3.4 ± 0.1h
L-Leucine	11.1 ± 0.6b	8.1 ± 0.6c	5.3 ± 0.2d	22.8 ± 0.4a	13.5 ± 2.4b	19.6 ± 1.4a	5.3 ± 0.4d	21.0 ± 1.1a	3.0 ± 0.1e	5.1 ± 0.1d	3.5 ± 0.6e	3.2 ± 0.3e	5.3 ± 0.2d
L-Phenylalanine	12.2 ± 1.2e	31.4 ± 1.5b	17.8 ± 1.4d	21.6 ± 1.1c	7.2 ± 1.6f	21.3 ± 2.1c	5.0 ± 0.6g	51.8 ± 1.4a	23.2 ± 0.6c	17.1 ± 0.7d	4.6 ± 0.2g	11.1 ± 0.4e	3.8 ± 0.4h
L-Tryptophan	10.7 ± 1.1b	4.3 ± 0.9e	9.3 ± 0.9c	14.9 ± 1.0a	10.0 ± 2.1b	7.8 ± 1.1d	5.9 ± 0.2c	9.3 ± 0.6c	10.3 ± -0.9b	6.6 ± 0.3c	2.0 ± 0.1f	5.5 ± 0.1e	2.9 ± 0.1f
Σ AA	592.2d	1292.1a	579.5fd	864.7b	497.1fg	755.4c	321.4i	537.7ef	342.5i	455.0g	162.3j	397.9h	199.4j
ANOVA test ^†^	A								B				

^†^ Significant at *p* < 0.05; AA—amino acid; nd—not detected.

**Table 4:** In vitro biological activity as anti-oxidant capacity (ORAC, FRAP, ABTS) and anti-diabetic, anti-obesity, and anti-cholinergic activity of sprouts and microgreens.

Sample		Anti-Oxidant Capacity			Anti-Diabetic Activity		Anti-Obesity Activity	Anti-Cholinergic Activity	
		ORAC	FRAP	ABTS	α-Amylase	α-Glucosidase		AChE	BuChE
Sprouts	radish (*Raphanus sativus*)	4.5 ± 0.3c ^‡^	0.6 ± 0.1b	1.8 ± 0.2b	50.8 ± 4.8g	6.7 ± 0.7e	0.5 ± 0.0g	21.3 ± 2.4d	11.7 ± 1.2fg
	lentil (*Lens culinaris*)	4.4 ± 0.6c	0.1 ± 0.0g	2.0 ± 0.3a	88.4 ± 5.3h	15.9 ± 2.1g	1.5 ± 0.2h	10.1 ± 1.0fg	1.0 ± 0.3h
	black medick (*Medicago lupulina*)	2.5 ± 0.3d	0.1 ± 0.0h	0.8 ± 0.1e	4.9 ± 0.8b	0.6 ± 0.2a	0.1 ± 0.0bc	8.6 ± 0.6g	3.6 ± 0.2h
	broccoli (*Brassica oleracea* var. italica)	5.7 ± 0.3a	0.8 ± 0.1a	1.8 ± 0.4b	6.9 ± 0.5cd	51.0 ± 3.5h	0.4 ± 0.1f	98.0 ± 2.8a	92.0 ± 3.8a
	sunflower (*Helianthus annuus* L.)	2.4 ± 0.1d	0.2 ± 0.0f	0.6 ± 0.0f	9.8 ± 1.0e	3.2 ± 0.2cd	0.3 ± 0.0	22.0 ± 0.7d	9.7 ± 0.6g
	leek (*Allium porrum*)	1.1 ± 0.2g	0.1 ± 0.0gh	0.8 ± 0.1e	10.4 ± 0.5e	2.0 ± 0.3bc	0.3 ± 0.0e	12.8 ± 0.8ef	10.0 ± 1.2g
	beetroot (*Beta vulgaris*)	5.9 ± 0.5a	0.3 ± 0.0c	1.3 ± 0.2c	5.4 ± 0.2bc	2.0 ± 0.2bc	0.2 ± 0.0cd	22.7 ± 1.3d	14.3 ± 1.3fg
	mung beans (*Vigna radiata*)	1.1 ± 0.1fg	>0.01i	>0.01h	5.3 ± 0.3b	0.9 ± 0.3bc	0.2 ± 0.0d	14.2 ± 1.2e	3.0 ± a0.5h
Microgreens	kale (*Brassica oleracea*)	2.4 ± 0.2d	0.3 ± 0.0d	1.0 ± 0.2d	4.8 ± 0.3b	4.3 ± 0.6d	0.1 ± 0.0a	59.2 ± 3.6b	79.4 ± 2.5b
	radish (*Raphanus sativus*)	1.8 ± 0.2e	0.1 ± 0.0f	0.6 ± 0.2f	7.7 ± 0.6d	1.3 ± 0.2bc	0.2 ± 0.0d	9.0 ± a1.1g	16.4 ± 1.1de
	beetroot (*Beta vulgaris*)	5.3 ± 0.3b	0.2 ± 0.0e	0.2 ± 0.0g	2.9 ± 0.1a	1.4 ± 0.1bc	0.1 ± 0.0a	12.0 ± 0.8efg	8.3 ± 0.4g
	green peas *(Pisum sativum)*	1.2 ± 0.3fg	0.1 ± 0.0g	0.7 ± 0.1e	8.3 ± 0.5d	8.0 ± 0.1f	0.1 ± 0.0bc	52.5 ± 0.4c	19.6 ± 2.4d
	amaranth (*Amaranthus)*	1.4 ± 0.2f	0.1 ± 0.0f	0.6 ± 0.1f	12.5 ± 0.9f	4.3 ± 0.2d	0.3 ± 0.0e	50.5 ± 1.4c	54.9 ± 3.5c
ANOVA test ^†^									
Sprouts		A	A	A	B	B	B	B	B
Microgreens		B	B	B	A	A	A	A	A

^†^ Significant at *p* < 0.05; ^‡^ values (mean of three replications) followed by the same letter within the same column were not significantly different (*p* < 0.05) according to Tukey’s least significant difference test; antioxidant capacity as mMol Trolox/100 g fw; anti-diabetic and anti-obesity as IC50 (mg/mL); anti-cholinergic activity as % of inhibition.

## References

[1] Samotyja U., Zdziebłowski T., Szlachta M. (2012). Właściwości przeciwutleniają ce naturalnych ekstraktów polifenolowych z wybranych roślin w układach modelowych. Nauk. Technol. Żywność..

[2] Baenas N., Gómez-Jodar I., Moreno D.A., García-Viguera C., Periago P.M. (2017). Broccoli and radish sprouts are safe and rich in bioactive phytochemicals. Postharvest Biol. Technol..

[3] Silva L.R., Pereira M.J., Azevedo J., Gonçalves R.F., Valentão P., de Pinho P.G., Andrade P.B. (2013). *Glycine max* L. Merr., *Vigna radiata* L. and *Medicago sativa* L. sprouts: A natural source of bioactive compounds. Food Res. Int..

[4] Kyriacou M.C., El-Nakhel C., Graziani G., Pannico A., Soteriou G.A., Giordano M., Ritieni A., De Pascale S., Rouphael Y. (2019). Functional quality in novel food sources: Genotypic variation in the nutritive and phytochemical composition of thirteen microgreens species. Food Chem..

[5] Cevallos-Casals B.A., Cisneros-Zevallos L. (2010). Impact of germination on phenolic content and antioxidant activity of 13 edible seed species. Food Chem..

[6] Kyriacou M.C., Rouphael Y., Di Gioia F., Kyratzis A., Serio F., Renna M., De Pascale S., Santamaria P. (2016). Micro-scale vegetable production and the rise of microgreens. Trends Food Sci. Technol..

[7] Treadwell D.D., Hochmuth R., Landrum L., Laughlin W. (2010). Microgreens: A new specialty crop. EDIS.

[8] Sun J., Xiao Z., Lin L., Lester G.E., Wang Q., Harnyl J.M., Chen P. (2008). Profiling polyphenols in five *Brassica* species microgreens by UHPLC-PDA-ESI/HRMS. Bone.

[9] Xiao Z., Codling E.E., Luo Y., Nou X., Lester G.E., Wang Q. (2016). Microgreens of Brassicaceae: Mineral composition and content of 30 varieties. J. Food Compos. Anal..

[10] Choe U., Yu L.L., Wang T.T.Y. (2018). The science behind microgreens as an exciting new food for the 21st Century. J. Agric. Food Chem..

[11] Villaño D., López-Chillón M.T., Zafrilla P., Moreno D.A. (2019). Bioavailability of broccoli sprouts in different human overweight populations. J. Funct. Foods.

[12] Xiao Z., Lester G.E., Luo Y., Wang Q. (2012). Assessment of vitamin and carotenoid concentrations of emerging food products: Edible microgreens. J. Agric. Food Chem..

[13] Kim S.J., Maeda T., Sarker M.Z.I., Takigawa S., Matsuura-Endo C., Yamauchi H., Mukasa Y., Saito K., Hashimoto N., Noda T. (2007). Identification of anthocyanins in the sprouts of buckwheat. J. Agric. Food Chem..

[14] Turkiewicz I.P., Wojdyło A., Tkacz K., Nowicka P., Hernández F. (2019). Antidiabetic, anticholinesterase and antioxidant activity vs. terpenoids and phenolic compounds in selected new cultivars and hybrids of artichoke cynara scolymus L.. Molecules.

[15] Tkacz K., Wojdyło A., Turkiewicz I.P., Bobak Ł., Nowicka P. (2019). Anti-oxidant and anti-enzymatic activities of sea buckthorn (*Hippophaë rhamnoides* L.) fruits modulated by chemical components. Antioxidants.

[16] Samuoliene G., Brazaityte A., Jankauskiene J., Viršile A., Sirtautas R., Novičkovas A., Sakalauskiene S., Sakalauskaite J., Duchovskis P. (2013). LED irradiance level affects growth and nutritional quality of *Brassica* microgreens. Cent. Eur. J. Biol..

[17] Marton M., Mandoki Z., Caspo J., Caspo-Kiss Z., Marton M., Mándoki Z., Marton M., Mandoki Z., Caspo J., Caspo-Kiss Z. (2010). The role of sprouts in human nutrition. A review. Aliment. Hungarian Univ. Transylvania.

[18] Cefola M., Pace B. (2015). Application of Oxalic Acid to Preserve the Overall Quality of Rocket and Baby Spinach Leaves during Storage. J. Food Process. Preserv..

[19] Ruíz-Jiménez J.M., Zapata P.J., Serrano M., Valero D., Martínez-Romero D., Castillo S., Guillén F. (2014). Effect of oxalic acid on quality attributes of artichokes stored at ambient temperature. Postharvest Biol. Technol..

[20] Riga P., Benedicto L., Gil-Izquierdo Á., Collado-González J., Ferreres F., Medina S. (2019). Diffuse light affects the contents of vitamin C, phenolic compounds and free amino acids in lettuce plants. Food Chem..

[21] Waterland N.L., Moon Y., Tou J.C., Kim M.J., Pena-Yewtukhiw E.M., Park S. (2017). Mineral content differs among microgreen, baby leaf, and adult stages in three cultivars of kale. Hort. Sci..

[22] Weber C.F. (2017). Broccoli Microgreens: A mineral-rich crop that can diversify food systems. Front. Nutr..

[23] Xu M.J., Dong J.F., Zhu M.Y. (2005). Effects of germination conditions on ascorbic acid level and yield of soybean sprouts. J. Sci. Food Agric..

[24] Samuolienė G., Viršilė A., Brazaitytė A., Jankauskienė J., Sakalauskienė S., Vaštakaitė V., Novičkovas A., Viškelienė A., Sasnauskas A., Duchovskis P. (2017). Blue light dosage affects carotenoids and tocopherols in microgreens. Food Chem..

[25] Alrifai O., Hao X., Marcone M.F., Tsao R. (2019). Current review of the modulatory effects of LED lights on photosynthesis of secondary metabolites and future perspectives of microgreen vegetables. J. Agric. Food Chem..

[26] Brazaityte A., Sakalauskiene S., Samuoliene G., Jankauskiene J., Viršile A., Novičkovas A., Sirtautas R., Miliauskiene J., Vaštakaite V., Dabašinskas L. (2015). The effects of LED illumination spectra and intensity on carotenoid content in Brassicaceae microgreens. Food Chem..

[27] O’Neill M.E., Carroll Y., Corridan B., Olmedilla B., Granado F., Blanco I., Van den Berg H., Hininger I., Rousell A.-M., Chopra M. (2001). A European carotenoid database to assess carotenoid intakes and its use in a five-country comparative study. Br. J. Nutr..

[28] Mir S.A., Shah M.A., Mir M.M. (2017). Microgreens: Production, shelf life, and bioactive components. Crit. Rev. Food Sci. Nutr..

[29] Wojdyło A., Nowicka P., Bąbelewski P. (2018). Phenolic and carotenoid profile of new goji cultivars and their anti-hyperglycemic, anti-aging and antioxidant properties. J. Funct. Foods.

[30] Silva B.M., Casal S., Andrade P.B., Seabra R.M., Oliveira M.B.P.P., Ferreira M.A. (2004). Free Amino acid composition of quince (*Cydonia oblonga* Miller) fruit (pulp and peel) and jam. J. Agric. Food Chem..

[31] Tarasevičiene Ž., Danilčenko H., Jariene E., Paulauskiene A., Gajewski M. (2009). Changes in some chemical components during germination of broccoli seeds. Not. Bot. Horti Agrobot. Cluj-Napoca.

[32] Collado-González J., Cruz Z.N., Medina S., Mellisho C.D., Rodríguez P., Galindo A., Egea I., Romojaro F., Ferreres F., Torrecillas A. (2014). Effects of water deficit during maturation on amino acids and jujube fruit eating quality. Maced. J. Chem. Chem. Eng..

[33] Kobus-Cisowska J., Szymanowska D., Maciejewska P., Kmiecik D., Gramza-Michałowska A., Kulczyński B., Cielecka-Piontek J. (2019). In vitro screening for acetylcholinesterase and butyrylcholinesterase inhibition and antimicrobial activity of chia seeds (*Salvia hispanica*). Electron. J. Biotechnol..

[34] Nowicka P., Wojdyło A. (2019). Anti-hyperglycemic and anticholinergic effects of natural antioxidant contents in edible flowers. Antioxidants.

[35] Kanetro B. (2018). Amino acid profile of soybean (*Glicine max*) sprout protein for determining insulin stimulation amino acids. Int. Food Res. J..

[36] Bahadoran Z., Mirmiran P., Hosseinpanah F., Rajab A., Asghari G., Azizi F. (2012). Broccoli sprouts powder could improve serum triglyceride and oxidized LDL/LDL-cholesterol ratio in type 2 diabetic patients: A randomized double-blind placebo-controlled clinical trial. Diabetes Res. Clin. Pract..

[37] Wojdyło A., Nowicka P., Grimalt M., Legua P., Almansa M.S., Amorós A., Carbonell-Barrachina Á.A., Hernández F. (2019). Polyphenol compounds and biological activity of caper (*Capparis spinosa* L.) flowers buds. Plants.

[38] Wojdyło A., Jáuregui P.N.N., Carbonell-Barrachina Á.A., Oszmiański J., Golis T. (2013). Variability of phytochemical properties and content of bioactive compounds in *Lonicera caerulea* L. var. kamtschatica Berries. J. Agric. Food Chem..

[39] Wojdyło A., Nowicka P., Carbonell-Barrachina Á.A., Hernández F. (2016). Phenolic compounds, antioxidant and antidiabetic activity of different cultivars of *Ficus carica* L. fruits. J. Funct. Foods.

[40] Wojdyło A., Oszmiański J., Bielicki P. (2013). Polyphenolic composition, antioxidant activity, and polyphenol oxidase (PPO) activity of quince (*Cydonia oblonga* Miller) varieties. J. Agric. Food Chem..

[41] Ou B., Huang D., Hampsch-Woodill M., Flanagan J.A., Deemer E.K. (2002). Analysis of antioxidant activities of common vegetables employing oxygen radical absorbance capacity (ORAC) and ferric reducing antioxidant power (FRAP) assays: A comparative study. J. Agric. Food Chem..

[42] Benzie I.F.F., Strain J.J. (1996). The ferric reducing ability of plasma (FRAP) as a measure of “antioxidant power”: The FRAP assay. Anal. Biochem..

[43] Re R., Pellegrini N., Proteggente A., Pannala A., Yang M., Rice-Evans C. (1999). Antioxidant activity applying an improved ABTS radical cation decolorization assay. Free Radic. Biol. Med..

